# Executive function as a generalized determinant of psychopathology and functional outcome in school-aged autism spectrum disorder: a case-control study

**DOI:** 10.1017/S0033291722001787

**Published:** 2023-07

**Authors:** Oscar W. H. Wong, Ran Barzilay, Angela M. W. Lam, Sandra Chan, Monica E. Calkins, Raquel E. Gur, Ruben C. Gur

**Affiliations:** 1 Department of Psychiatry, The Chinese University of Hong Kong, Hong Kong; 2 Neurodevelopment and Psychosis Section, Department of Psychiatry, Perelman School of Medicine, University of Pennsylvania, Philadelphia, Pennsylvania, USA; 3 Lifespan Brain Institute (LiBI), Children's Hospital of Philadelphia and Penn Medicine, Philadelphia, Pennsylvania, USA; 4 Department of Child and Adolescent Psychiatry and Behavioral Sciences, Children's Hospital of Philadelphia and Penn Medicine, Philadelphia, Pennsylvania, USA

**Keywords:** Autism spectrum disorder, executive function, neurocognition, psychiatric comorbidity, psychopathology, social cognition

## Abstract

**Background:**

Individuals with autism spectrum disorder (ASD) are challenged not only by the defining features of social-communication deficits and restricted repetitive behaviors, but also by a myriad of psychopathology varying in severity. Different cognitive deficits underpin these psychopathologies, which could be subjected to intervention to alter the course of the disorder. Understanding domain-specific mediating effects of cognition is essential for developing targeted intervention strategies. However, the high degree of inter-correlation among different cognitive functions hinders elucidation of individual effects.

**Methods:**

In the Philadelphia Neurodevelopmental Cohort, 218 individuals with ASD were matched with 872 non-ASD controls on sex, age, race, and socioeconomic status. Participants of this cohort were deeply and broadly phenotyped on neurocognitive abilities and dimensional psychopathology. Using structural equation modeling, inter-correlation among cognitive domains were adjusted before mediation analysis on outcomes of multi-domain psychopathology and functional level.

**Results:**

While social cognition, complex cognition, and memory each had a unique pattern of mediating effect on psychopathology domains in ASD, none had significant effects on the functional level. In contrast, executive function was the only cognitive domain that exerted a generalized negative impact on every psychopathology domain (*p* factor, anxious-misery, psychosis, fear, and externalizing), as well as functional level.

**Conclusions:**

Executive function has a unique association with the severity of comorbid psychopathology in ASD, and could be a target of interventions. As executive dysfunction occurs variably in ASD, our result also supports the clinical utility of assessing executive function for prognostic purposes.

## Introduction

Autism spectrum disorder (ASD) is a heterogeneous neurodevelopmental disorder. In addition to the core defining features of social communication deficits and restricted and repetitive behaviors (RRB), comorbid psychopathology is common in children with ASD (Simonoff et al., 2008). Comorbidities include anxiety disorders, attention-deficit/hyperactivity disorder (ADHD), and disruptive, impulse control disorders. Rarer syndromes such as bipolar disorders and schizophrenia spectrum disorders also occur at a higher rate in ASD than in the general population (Selten, Lundberg, Rai, & Magnusson, 2015). With the onset is usually at school-age, these psychiatric comorbidities further aggravate the overall burden on ASD individual's academic and social functioning, resulting in substantial detrimental effects along the developmental trajectory (Chiang & Gau, 2016). A recent meta-analysis identified unmodifiable factors such as age, sex, and intelligence as predictors of psychiatric comorbidities in ASD, however, a significant amount of unexplained variance remains (Lai et al., 2019). Hence, it is clinically relevant to identify other contributors, especially those that could be improved with intervention, such as the possible mediating factor of neurocognitive abilities in ASD.

As social communication difficulties constitute one of the two core features of ASD, the degree of social cognitive deficits translates directly into clinical severity and hence level of functioning (Bishop-Fitzpatrick, Mazefsky, Eack, & Minshew, 2017). Additionally, a range of cognitive dysfunctions is evident from an early age (Brunsdon & Happé, 2014; Pellicano, Gibson, Maybery, Durkin, & Badcock, 2005; Shalom, 2003; Tager-Flusberg & Joseph, 2003) and may act in concert to mediate behavioral and coping difficulties, resulting in externalizing and internalizing problems. A neuroimaging study revealed a shared white matter organization between ASD and ADHD at the corpus callosum, a region that underpins multiple cognitive processes (Aoki et al., 2017). This provides a biological basis for the speculation that psychiatric comorbidities in ASD could be contributed by cognitive dysfunctions. Understanding how individual cognitive domains contribute to psychopathology is important to inform the development of targeted interventions.

Among the range of cognitive domains, executive function (EF) may be a unique mediator of psychopathologies and functional outcomes in ASD. It has been well-established that ASD individuals have weaker EF when compared with typically developing individuals (Demetriou et al., 2018). Not merely an epiphenomenon, EF deficits negatively impact quality of life (de Vries & Geurts, 2015) and adaptive functioning (Pugliese et al., 2016) in ASD and contribute to theory of mind (ToM) impairments (Long, Horton, Rohde, & Sorace, 2018) and RRB (Iversen & Lewis, 2021). Yet, the degree of executive dysfunction varies between ASD individuals (Geurts, Sinzig, Booth, & Happé, 2014). This heterogeneity is intriguing, since EF, as a set of higher-order cognitive functions, could compensate for other cognitive deficits in neurodevelopmental disorders through recruitment of other brain systems (Johnson, 2012). Potentially acting as both risk and protective factors, EF may therefore contribute to diverse functional outcomes and ranges of comorbid psychopathology in ASD (Lai et al., 2019; Steinhausen, Mohr Jensen, & Lauritsen, 2016).

Studies have demonstrated that executive dysfunction in ASD could be related to psychopathology such as anxiety (Hollocks et al., 2014), depressive disorders (Wallace et al., 2016), aggressive behaviors (Lawson et al., 2015), and ADHD (Craig et al., 2016). However, as EF acts synergistically with other cognitive domains in daily functioning, concurrent examination of the entire range of cognitive functioning is needed to disentangle the complex interactive patterns and inter-relatedness among cognitive abilities and psychopathology. Furthermore, existing studies typically focus on only one to two types of psychiatric comorbidities, yet in real life, individuals with ASD may experience multiple psychiatric disorders with overlapping symptoms (Simonoff et al., 2008), alongside subthreshold symptoms (Caamaño et al., 2013). Rarer comorbidities such as schizophrenia and affective psychosis also warrant investigation given the severe disruption of well-being and everyday functioning that can ensue. Therefore, in the present study, using structural equation modeling (SEM), we investigated the interactions between neurocognitive abilities and dimensional psychopathology, both deeply and broadly phenotyped in a community-derived sample that included individuals with ASD, to address the research question of how different neurocognitive domains would be associated with comorbid psychopathology and functional level in school-aged ASD. We hypothesized that, after controlling for the inter-correlation between different cognitive domains, EF has a unique association with psychopathology and functional level in ASD.

## Methods and materials

### The Philadelphia Neurodevelopmental Cohort

Participants were from the Philadelphia Neurodevelopmental Cohort (PNC), a collaboration between the Children's Hospital of Philadelphia (CHOP) and the Brain Behavior Laboratory of the University of Pennsylvania (Calkins et al., 2015). The PNC recruited children and young adults who were (1) ages of 8–21 years, (2) ambulatory in stable health, (3) proficient in English, (4) had cognitive abilities to participate in study procedures, and (5) with no significant physical conditions or developmental delay impairing motility or cognition (e.g. paresis, palsy, or intellectual disability) from a large pool of children (*N* = 50 293) previously genotyped as part of a genomic study in the CHOP health care network. Of note, participants were recruited from pediatric rather than psychiatric clinics, and thus the sample was not enriched for those seeking psychiatric services. Participants were excluded if they did not meet enrollment criteria or could not be contacted. The PNC comprised of 9498 youths that were racially (56% Caucasian, 33% African American, and 11% others) and socioeconomically diverse, with a socioeconomic status (SES) score calculated based on the participant's geocoded neighborhood data of residents in poverty, marital status, median family income, and crime rates. Higher scores indicate better SES (Moore et al., 2016). The clinical phenotype assessment was administered in English to collateral informants including caregivers or legal guardians of probands aged 8–10; to both probands and collateral informants for those aged 11–17; and to probands only for those aged 18–21. Participants and their guardians (for participants <18 years) provided written informed consent or assent. Inclusion criteria for collaterals included proficiency in English, as determined by telephone screening during the initial recruitment call. All procedures were approved by the University of Pennsylvania and the Children's Hospital of Philadelphia Institutional Review Boards.

### Identification of ASD diagnoses and matched controls

As a part of the PNC protocol, probands (participants aged 11–21) or their parents (participants under 18) were asked whether they/their child had ever been diagnosed with autism, pervasive developmental disorder, or Asperger's syndrome (i.e. ASD). From the entire PNC cohort, 291 cases reported ASD diagnoses. To verify these ASD diagnoses through independent documentation, a search of participants' CHOP electronic health record (EHR) was conducted, yielding a total of 202 participants with both PNC-reported and CHOP EHR documentation of their ASD diagnoses. Thereafter, another search of the entire PNC population was done for the presence of ICD codes in the autism spectrum [autism, pervasive developmental disorder (PDD), PDD-not otherwise specified, ASD]. This search yielded an additional 16 cases of ASD, who were also verified by additional documentation of ASD in their EHR. Participants were classified in the ASD group if they fulfilled the criteria of (1) documentation of ASD diagnosis in any health care provider's note or visit summary; and (2) presence of ICD code of ASD diagnoses. Participants who endorsed ASD diagnoses yet did not have any confirming documentation indicating ASD in their EHR were excluded (*n* = 89). Thus, the ASD group comprised 218 participants. Comparison subjects were identified from the remaining PNC population (*n* = 9280) using the R package ‘Matchlt’ (Ho, Imai, King, & Stuart, 2011) and matched to the ASD group based on age, sex, SES, and race in a 4:1 ratio, resulting in 872 matched non-ASD comparison subjects.

### Neurocognitive phenotyping

Cognitive assessment was conducted using the Penn Computerized Neurocognitive Battery (CNB), a 1-hour computerized battery including 12 tasks in the following cognitive domains: social cognition (emotion identification, emotion intensity differentiation, and age differentiation), complex cognition (language reasoning, non-verbal reasoning, and spatial ability), episodic memory (verbal, spatial, and face), and EF (attention, working memory, and abstraction and mental flexibility). The CNB was validated as a reliable and robust measure of cognitive function across ages in both healthy and psychiatric samples (Almasy et al., 2008; Irani et al., 2012; Moore, Reise, Gur, Hakonarson, & Gur, 2015). For each task, accuracy and speed were measured and *z*-transformed, and an efficiency score was calculated by averaging the accuracy and speed *z*-scores. The cognitive performance was then evaluated by factor analysis, delineating the loadings of the tasks into four domains: EF, episodic memory, complex cognition, and social cognition (Moore et al., 2015). To ensure that the participants could complete the battery, an estimated intelligence (IQ) of ⩾70 with the Wide Range Achievement Test (WRAT-4) Reading subscale (Wilkinson & Robertson, 2006) was used as a cut-off, as reading ability is relatively resistant to brain insult and is suitable for estimating premorbid IQ (Kareken, Gur, & Saykin, 1995). The WRAT-4 Reading subscale score was converted into an age-standardized score (mean = 100 and standard deviation = 15) to provide an estimated IQ score.

### Psychopathology and clinical phenotyping

Psychopathology symptoms were evaluated by trained and supervised assessors using a structured screening interview GOASSESS (Grand Opportunity Assessment), developed based on the Kiddie Schedule for Affective Disorders and Schizophrenia (K-SADS) (Kaufman et al., 1997) with additional screening questions and dimensional ratings of distress and impairments associated with symptoms in each diagnostic section, and less restrictions to complete the diagnostic modules (Calkins et al., 2015). Lifetime psychiatric diagnoses were determined if symptoms were endorsed with frequency and duration approximating DSM-IV disorder or episode criteria, with significant distress or impairments. To evaluate the youths' overall functioning, the Children's Global Assessment Scale (C-GAS) (Shaffer et al., 1983) was used, considering their behavior in different environments, relationships, and disturbances associated with psychiatric symptoms. Higher scores in C-GAS indicates better functioning, with scores >70 indicating normal functioning (Bird et al., 1990). To allow the dimensional quantification of psychopathology, psychopathology factor scores were generated using itemwise responses (at the symptom level) from clinical interviews across all assessed psychopathology domains. Based on previous factor analyses with responses from the informants (age 8–10) and the participants (age 11–21), the 112 item-level symptoms from the GOASSESS were reduced into five orthogonal dimensions of psychopathology, including four specific factors of anxious-misery (depressed mood, anxiety, and obsessive-compulsive symptoms), psychosis, externalizing behaviors, and fear (specific phobias such as agoraphobia and social phobia), as well as an overall psychopathology factor (i.e. *p* factor) (Shanmugan et al., 2016). The *p* factor integrates multiple symptom domains including internalizing and externalizing symptoms, and is well suited for mapping psychopathology liabilities in childhood and adolescence (Allegrini et al., 2020).

### Statistical analysis

Data management and statistical analyses were performed using the R 4.0.3 statistical environment (R Studio, Version 1.4). Missing data for neurocognitive and psychopathology scores were handled with multiple imputations using the R package ‘Amelia’. The variables to be used were included in the imputation model. Given the large sample size, 10 imputations were used to generate a combined imputed dataset for subsequent analyses. Descriptive statistics including demographics and clinical characteristics were examined with χ^2^ tests for categorical data and independent *t* tests or Mann–Whitney's *U* test for continuous data upon normality checking. Comparisons of neurocognitive efficiency, psychopathology, and C-GAS between ASD and non-ASD participants were analyzed using multivariate analysis of covariance (MANCOVA), preceded by correlation analysis for checking potential correlations.

For the main analyses, SEM was conducted to examine mediated relationships between the measured variables using Mplus, Version 7 (Muthén & Muthén, 2012). Three models were fitted to test the predictive effects of different neurocognitive domains on psychopathological and functional outcomes. Models 1 and 2 examined the predictive relationships between ASD, neurocognitive efficiency, and the *p* factor (model 1), and the four factors of psychopathology (anxious-misery, psychosis, externalizing, and fear) (model 2) respectively. Model 3 examined the predictive relationships between ASD, neurocognitive efficiency, and general functional level (C-GAS). Covariates were adjusted for all three models upon correlation checks. Mediation analysis was conducted to assess the mediating pathways in the final step.

Model fit and path estimates (*β* coefficients) for the models were estimated with Maximum Likelihood (MLR) estimation with robust standard errors to accommodate potential non-normality in the variables. Model fit indices included χ^2^ statistics with its degree of freedom (df) and *p* values, the root mean square error of approximation (RMSEA) (Steiger, 1990) and its 90% confidence interval (CI), the comparative fit index (CFI) (Bentler, 1990), and the standardized root mean square residual (SRMR) test. RMSEA ⩽ 0.05 and ⩽0.08 indicate a good model fit and reasonable model fit, respectively. CFI ⩾ 0.90 suggest a reasonably good model fit, although a CFI ⩾ 0.95 is preferable (Kline, 2015). SRMR ⩽ 0.08 is generally considered a good fit of model (Hu & Bentler, 1999). The 95% bias-corrected bootstrapping and the Benjamini–Hochberg's false discovery rate (FDR) method to control for multiple comparisons were applied to all path effect estimations in mediation analysis, with standardized estimates and CIs reported.

## Results

### Participants' demographics, neurocognitive and psychopathological phenotyping, and preliminary analysis of covariates

There were 171 males and 47 females in the ASD group (mean age = 12.27, s.d. = 3.02) and 684 males and 188 females in the non-ASD group (mean age = 12.33, s.d. = 3.30). The two groups were not significantly different in race, participants' and parental educational level, estimated IQ, and SES. Functional level was significantly lower in the ASD group (online Supplementary Table S1). Correlation analysis was conducted to identify potential covariates (online Supplementary Table S2). As sex, age, race, and SES correlate with neurocognitive efficiencies and psychopathology factors and could confound outcome measurements, they were controlled as covariates in the subsequent MANCOVA and SEM.

Two separate MANCOVAs were conducted using ASD diagnosis as the fixed factor, with neurocognitive efficiencies and psychopathology factors as the dependent variables respectively. Results revealed significant effects of ASD on both neurocognitive efficiencies [Pillai's Trace = 0.057, *F*_(4, 1068)_ = 16.136, *p* < 0.001, *η*^2^ = 0.014] and psychopathology factors [Pillai's Trace = 0.111, *F*_(4, 1064)_ = 26.659, *p* < 0.001, *η*^2^ = 0.043]. The univariate *F* tests showed significantly lower efficiency in all neurocognitive domains and significantly higher scores in all psychopathology factors in ASD participants, when compared with non-ASD participants. The neurocognitive and psychopathology profiles of ASD and non-ASD participants are summarized in Table 1 and illustrated in online Supplementary Fig. S1.

**Table 1.** Summary of MANCOVA on neurocognitive efficiencies and psychopathology factors between ASD and non-ASD groups

Neurocognitive efficiencies	Multivariate tests							
			Value	*F*	df1	df2	*p*	*η*^2^
	ASD	Pillai's trace	0.057	16.136	4	1068	< 0.001	0.014
	Univariate tests							
		Dependent variable	SS	df	MS	*F*	*p*	*η*^2^
	ASD	Social cognition	35.582	1	35.582	33.557	<0.001	0.028
		Complex reasoning	5.985	1	5.985	6.671	0.010	0.006
		Memory	48.595	1	48.595	48.374	<0.001	0.043
		Executive unction	19.117	1	19.117	15.148	<0.001	0.013
	Residuals	Social cognition	1135.628	1071	1.060			
		Complex reasoning	960.872	1071	0.897			
		Memory	1075.890	1071	1.005			
		Executive function	1351.552	1071	1.262			
Psychopathology factors	Multivariate tests							
			Value	*F*	df1	df2	*p*	*η*^2^
	ASD	Pillai's trace	0.111	26.659	5	1064	<0.001	0.043
	Univariate tests							
		Dependent variable	SS	df	MS	*F*	*p*	*η*^2^
	ASD	Anxious-misery	82.533	1	82.533	88.761	<0.001	0.076
		Psychosis	70.109	1	70.109	79.482	<0.001	0.068
		Externalizing	90.931	1	90.931	96.731	<0.001	0.080
		Fear	57.357	1	57.357	64.540	<0.001	0.056
		*p* factor	62.319	1	62.319	69.755	<0.001	0.060
	Residuals	Anxious-misery	993.056	1068	0.930			
		Psychosis	942.064	1068	0.882			
		Externalizing	1003.961	1068	0.940			
		Fear	949.130	1068	0.889			
		*p* factor	954.138	1068	0.893			

*Note.* Results were controlled for sex, age, race, and socioeconomic status.

### Structural equation modeling

SEMs were first conducted to examine the mediating pathways, followed by mediation analyses to test for specific indirect path effects. *p* factor score (model 1), four psychopathology factors (model 2), and C-GAS (model 3) were regressed on the predictive variables (diagnosis of ASD) and all mediator variables (the four domains of neurocognitive efficiencies).

**Fig. 1.**
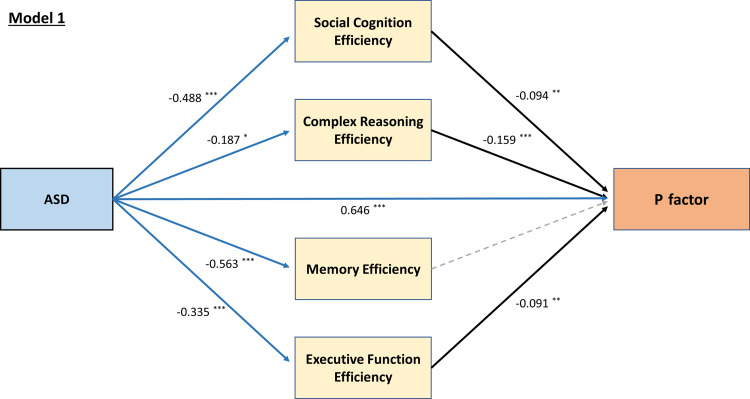
Structural regression model for the effect of ASD on the *p* factor, mediated by neurocognitive function efficiencies. Presented estimates are *β* coefficients, with statistically significant paths shown in solid lines. *p < 0.05, **p < 0.01, ***p < 0.001.

**Fig. 2.**
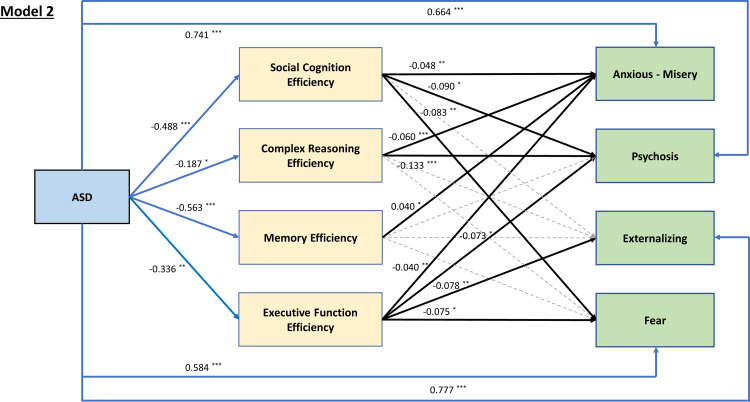
Structural regression model for the effect of ASD on the four psychopathology factors, mediated by neurocognitive function efficiencies. Presented estimates are *β* coefficients, with statistically significant paths shown in solid lines. *p < 0.05, **p < 0.01, ***p < 0.001.

**Fig. 3.**
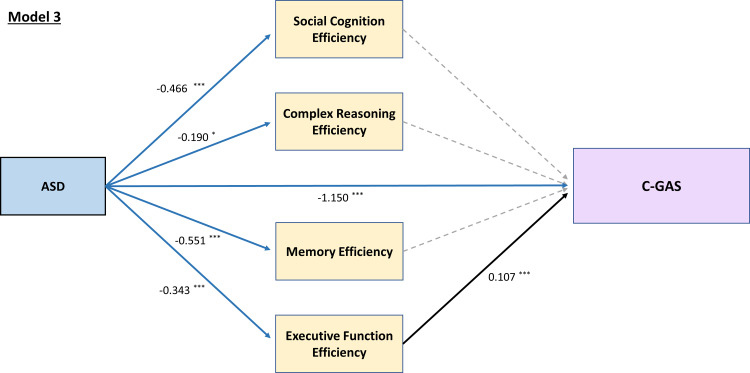
Structural regression model for the effect of ASD on functional level, as measured by the Children's Global Assessment Scale (C-GAS), mediated by neurocognitive function efficiencies. Presented estimates are *β* coefficients, with statistically significant paths shown in solid lines. *p < 0.05, **p < 0.01, ***p < 0.001.

Model 1 demonstrated reasonably good model fit [χ^2^ (5) = 28.525, *p* < 0.001; CFI = 0.987, RMSEA = 0.066, 90% CI 0.044–0.090, SRMR = 0.015]. Path estimates (standardized coefficients) of model 1 were illustrated in Fig. 1. Mediation analysis revealed a significant direct effect of ASD on the *p* factor [*β* = 0.646, *p* < 0.001], with social cognition [*β* = 0.042, *p* = 0.008], complex reasoning [*β* = 0.029, *p* = 0.042], and EF [*β* = 0.026, *p* = 0.033] as significant indirect pathways. Similarly, model 2 had a good model fit [χ^2^ (16) = 64.925, *p* < 0.001; CFI = 0.990, RMSEA = 0.053, 90% CI 0.040–0.067, SRMR = 0.018]. ASD had significant direct effects on all four psychopathology factors of anxious-misery [*β* = 0.741 (*p* < 0.001)], psychosis [*β* = 0.664 (*p* < 0.001)], externalizing [*β* = 0.777 (*p* < 0.001)], and fear [*β* = 0.584 (*p* < 0.001)]. Subsequent analyses showed differential mediating patterns of individual neurocognitive domain on psychopathology: social cognition significantly mediated the relationships between ASD and anxious-misery [*β* = 0.22, *p* = 0.009], psychosis [*β* = 0.040, *p* = 0.013], and fear [*β* = 0.037, *p* = 0.017]; complex reasoning mediated the paths between ASD and anxious-misery [*β* = 0.011, *p* = 0.044], and psychosis [*β* = 0.025, *p* = 0.045]; whereas the mediating effect of memory efficiency was only significant between ASD and anxious-misery but in a negative direction [*β* = −0.022, *p* = 0.021], i.e. better memory efficiency in ASD predicts higher factor score of anxious-misery. EF was the only domain that is a significant mediator of ASD and all four psychopathology factors of anxious-misery [*β* = 0.012, *p* = 0.036], psychosis [*β* = 0.021, *p* = 0.049], externalizing [*β* = 0.024, *p* = 0.037], and fear [*β* = 0.022, *p* = 0.043] (Fig. 2). Model 3 also had a good fit [χ^2^ (4) = 10.974, *p* = 0.027; CFI = 0.996, RMSEA = 0.040, 90% CI 0.012–0.069, SRMR = 0.011], with ASD significantly predicting C-GAS directly [*β* = −1.150, *p* < 0.001]. EF efficiency was the only significant mediating path [*β* = −0.031, *p* = 0.023] between ASD and the C-GAS (total indirect effect: *β* = −0.051, *p* = 0.032) (Fig. 3). Results of the goodness of fit statistics and the mediation path estimates of the SEMs are summarized in online Supplementary Table S3 and Table 2 respectively.

**Table 2.** Mediation path estimates of the three structural equation models

Model 1	*p* factor	
	*β* [95% CI]	*p*
Direct effect		
ASD	0.646 [0.490–0.794]	<0.001
Indirect effect		
ASD via social cognition efficiency	0.042 [0.016–0.077]	0.008
ASD via complex reasoning efficiency	0.029 [0.002–0.060]	0.042
ASD via memory efficiency	0.007 [−0.026 to 0.042]	0.691
ASD via executive function efficiency	0.026 [0.008–0.052]	0.033

Model 2	Anxious-misery		Psychosis		Externalizing		Fear	
	*β* [95% CI]	*p*	*β* [95% CI]	*p*	*β* [95% CI]	*p*	*β* [95% CI]	*p*
Direct effect								
ASD	0.741 [0.577–0.893]	<0.001	0.664 [0.511–0.800]	<0.001	0.777 [0.621–0.920]	<0.001	0.584 [0.734–0.584]	<0.001
Indirect effect								
ASD via social cognition efficiency	0.022 [0.008–0.038]	0.009	0.040 [0.013–0.074]	0.013	0.009 [−0.020 to 0.039]	0.653	0.037 [0.009–0.067]	0.017
ASD via complex reasoning efficiency	0.011 [0.001–0.025]	0.044	0.025 [0.002–0.053]	0.045	0.020 [−0.002 to 0.046]	0.074	0.019 [−0.001 to 0.042]	0.056
ASD via memory efficiency	−0.022 [−0.042 to −0.007]	0.021	0.019 [−0.012 to 0.053]	0.377	0.013 [−0.021 to 0.048]	0.552	0.025 [−0.013 to 0.062]	0.162
ASD via executive function efficiency	0.012 [0.003–0.023]	0.036	0.021 [0.005–0.045]	0.049	0.024 [0.005–0.051]	0.037	0.022 [0.004–0.047]	0.043

Model 3	C-GAS	
	*β* [95% CI]	*p*
Direct effect		
ASD	−1.150 [−1.291 to −1.009]	<0.001
Indirect effect		
ASD via social cognition efficiency	−0.013 [−0.046 to 0.020]	0.489
ASD via complex reasoning efficiency	−0.016 [−0.036 to 0.004]	0.196
ASD via memory efficiency	0.010 [−0.022 to 0.042]	0.655
ASD via executive function efficiency	−0.031 [−0.057 to −0.006]	0.023

*Note*. ASD, autism spectrum disorder; C-GAS, Children's Global Assessment Scale. Results were controlled for sex, age, race, and socioeconomic status, 95% bootstrap confidence interval, with Benjamini–Hochberg's FDR method corrected for multiplicity.

With the entire cohort of participants with normal IQ (⩾70), estimated IQ by WRAT-4 did not correlate with neurocognitive efficiencies and psychopathology factors. This is consistent with results of previous studies that showed reading ability being weaker at predicting performance in specific cognitive domains (Schretlen, Buffington, Meyer, & Pearlson, 2005), especially in average and above-average IQ populations (Diaz-Asper, Schretlen, & Pearlson, 2004). However, as IQ can exert a ceiling effect on neurocognitive performance, sensitivity analyses were carried out by including estimated IQ as an additional covariate in the MANCOVA and the three SEM models. Regarding the MANCOVA, the ASD group remained to have lower cognitive efficiencies and higher psychopathology (online Supplementary Table S4). For the SEM models, incorporating estimated IQ did not result in significant Δ*R*^2^ for all three models (models 1a–3a, online Supplementary Figs S2–S4) to suggest improvement of the model fit (online Supplementary Table S3). EF remained as the only neurocognitive domain that mediated the path between ASD and all psychopathology factors and C-GAS (online Supplementary Table S5).

## Discussion

### Psychopathology and neurocognition of the community-derived sample of ASD

In the present study, the community-derived group of ASD with normal IQ represented a spectrum of varying functional levels and encompassed the full range of psychopathology. Compared to non-ASD, the ASD population had poorer current global functioning characterized by higher severity in all four specific domains of psychopathology and overall psychopathology. Neurocognitive efficiencies were also poorer in the ASD group across all four cognitive domains. Of note, the effect sizes for all these comparisons were small, which aligns with the heterogeneity in the degree of cognitive impairment (Chen et al., 2019; Geurts et al., 2014) and prevalence of psychiatric comorbidities in ASD (Lai et al., 2019). Given that different cognitive functioning could be influenced by an overarching general factor such as global efficiency (Barbey, 2018), as reflected in the high degree of correlations among different domains within our sample (online Supplementary Table S2), the SEMs employed were adjusted for their inter-correlations to estimate specific mediating effects of individual cognitive domains on psychopathology.

### Social cognition, complex cognition, and memory differentially predict psychopathology factors, but not functional level

As a core deficit of ASD, poorer social cognitive functioning predicts the severity of the *p* factor, as well as specific factors of anxious-misery, fear, and psychosis. The severity of this core deficit may translate directly into difficulties in social situations (Bishop-Fitzpatrick et al., 2017), and result in mood disturbances and fearful responses. As captured by the social cognition tasks, difficulties in reading emotions, an ability fundamental to understanding others' intentions, were shown to be associated with proneness to psychosis at the population level (Germine & Hooker, 2011). Delusional ideas could result from misunderstanding others' emotions, intentions, and subsequent mentalization failure (Chung, Barch, & Strube, 2014), contributing to common subthreshold psychotic symptoms and experiences among ASD individuals (Kiyono et al., 2020).

On the other hand, complex cognition predicts specific factors of anxious-misery and psychosis. The tasks loaded into this domain measured fluid intelligence (Moore et al., 2015), which is strongly associated with the general *g* factor in intelligence (Reynolds & Keith, 2017). The relationship between intelligence and internalizing symptoms is not straightforward: anxiety and IQ were found to have a quadratic relationship, with the highest level of anxiety clustered in ASD individuals with borderline intellectual disability, while depression and IQ was found to be positively associated (Edirisooriya, Dykiert, & Auyeung, 2020; Service et al., 2020). In our current sample of normal IQ ASD, complex cognition predicted negatively the factor of anxious-misery, which was loaded by symptoms of generalized anxiety disorder, obsessive-compulsive disorder, and depression (Shanmugan et al., 2016). The negative association of complex cognition with the psychosis factor was coherent with the known interaction effect of IQ with genetic susceptibility to schizophrenia (Kendler, Ohlsson, Sundquist, & Sundquist, 2015). Intelligence also mediates the common ‘jumping to conclusions’ reasoning bias in schizophrenia (Tripoli et al., 2021).

For memory, mediation analyses suggested that better memory was predictive of more severe psychopathology in the anxious-misery factor. It has been observed that episodic memory correlates with self-awareness in autism (Toichi, 2008), and the self-awareness of multiple deficits of ASD may underpin anxiety symptoms (Edirisooriya et al., 2020).

It is worth noting that some results of the present study were incongruent with previous studies. For example, social functioning was reported to contribute to externalizing behaviors (Shea, Payne, & Russo, 2018), while another study found no association between social cognition and anxiety and depressive features (Hollocks et al., 2014). This discrepancy could be due to our control of the inter-correlations among different cognitive domains, such that some cognitive domains no longer stand out as unique mediating paths. Moreover, social functioning and cognition measured in previous studies were parent-reported and focused mostly on ToM skills, whereas our social cognition tasks focused mainly on emotional recognition and differentiation, hence the results are not directly comparable.

While these three domains of cognition (social cognition, complex cognition, and memory) all contributed differentially to the spectrum of psychopathology, none of them significantly determined functional outcomes (C-GAS) in ASD. This result, especially with regards to the complex cognition domain, challenges the common conceptualization of ‘high-functioning autism’, which posits ASD individuals with high IQ having neurotypical or even superior levels of functioning. Our results suggested that fluid intelligence may not be a unique determining factor of outcomes of ASD. This aligns with a recent study showing that IQ may be an imprecise marker for functional abilities in ASD without intellectual disabilities (Alvares et al., 2020).

### A generalized impact of executive function on psychopathology and functional outcome in ASD

EF encompasses separable constructs such as working memory, inhibitory control, planning, and switching (Miyake et al., 2000). However, as opposed to fractionated impairments, a recent meta-analysis supports a unitary executive dysfunction underpinned by brain connectivity aberrations in ASD (Demetriou et al., 2018). Accordingly, EF was considered as a unitary construct in the present study.

In the SEM models that controlled for the known predictive factors for comorbid psychopathology in ASD (i.e. age, sex, and exclusion of sub-normal IQ) (Lai et al., 2019), our results demonstrated the independent contribution of EF to all psychopathology factors and functional level. Executive dysfunction as a transdiagnostic risk factor for major psychiatric syndromes in youths has been well-demonstrated (Lynch, Sunderland, Newton, & Chapman, 2021). In developing children, executive dysfunction may serve as an intermediate phenotype that predicts later psychopathology through compromising skills of adaptation and conflicts resolution (Romer & Pizzagalli, 2021). Thus, the current study shows that ASD may not be exceptional. However, given that social cognition deficit is a core feature of ASD, it is intriguing that social cognition did not predict functional levels of ASD as EF did. This result echoed the postulation that the deficits or strengths of EF in ASD may accentuate or diminish the real-life impact of impairments conferred by other domains of cognitive deficits in neurodevelopmental disorders (Johnson, 2012). Taken together, EF could be an important prognostic factor in ASD warranting the clinical value of assessing and subtyping executive dysfunction in ASD (Geurts et al., 2014).

In a longitudinal study following ASD children from the age of two, diminished core features of ASD to subclinical level in early adulthood among ASD individuals without intellectual disabilities were predicted by early improvements in RRB symptoms and less hyperactive symptoms during childhood, but not verbal IQ and severity of social deficits at baseline (Anderson, Liang, & Lord, 2014). Both RRB and hyperactivity symptoms in ASD were shown to be mediated by underlying executive dysfunction (Iversen & Lewis, 2021; Lukito et al., 2017). Another longitudinal study revealed that EF at 5 years old predicted ToM skills and adaptive abilities 12 years later (Kenny, Cribb, & Pellicano, 2019), in line with findings that working memory and cognitive flexibility are needed for ToM development (Bock, Gallaway, & Hund, 2015; Lecce & Bianco, 2018) by holding and switching between thoughts and intentions of self and others. Hence, the potential compensatory mechanisms conferred by EF in ASD may have a long-term impact on their development.

### Executive function as a possible linkage between genetic risk of ASD and the diverse clinical outcome

From a biological perspective, genetic variants identified as conferring risks for ASD were highly pleiotropic, as they are also associated with the psychiatric comorbidities of ASD including ADHD, depressive disorders, and schizophrenia (Thapar & Rutter, 2020). While mechanisms of the pleiotropic effects remain elusive, the common variants identified have implied roles in neuronal functions and corticogenesis (Grove et al., 2019). EF, mediated mostly by the prefrontal cortex and its associated network (Alvarez & Emory, 2006), could be a common pathway between the genetic risks of ASD and its pleiotropic effects on diverse clinical phenotypes. This possibility is supported by a recent study showing that polygenic risk scores of ASD predict executive dysfunction (Torske et al., 2020). Our empirical observational findings therefore warrant further investigations into the underlying mechanism through large-scale longitudinal studies along neurocognitive and psychopathology development of ASD whilst incorporating genomic data (Searles Quick, Wang, & State, 2021). Given putative evidence suggesting that EF in ASD could be improved through training and neuromodulation techniques (Kenworthy et al., 2014; Rothärmel et al., 2019), there is pressing anticipation by patients, caregivers, and medical professionals alike for an intervention that could alter the longitudinal course of this chronic disabling disorder. Nonetheless, the design of any treatment trials on executive dysfunction in ASD would not be complete without a sufficient understanding of the pathophysiology.

### Limitations

There are several limitations in the present study. First, the cross-sectional nature of the study limits causal inferences, and longitudinal studies are needed to verify the effect of early development of neurocognition on later psychopathology, as the reverse of psychopathology impeding EF development was also found (Romer & Pizzagalli, 2021) and bi-directional effects should also be considered. Though the stability of EF during childhood (Polderman et al., 2007) to adolescence and young adulthood (Friedman et al., 2016) was demonstrated, less is known whether this is the same for ASD, hence the current cognitive functioning captured by CNB may not represent the cognitive ability before and during the onset of the lifetime history of psychiatric symptoms measured by GOASSESS. Second, processing speed, another common impairment in ASD (Oliveras-Rentas, Kenworthy, Roberson, Martin, & Wallace, 2012), was not specifically measured. The use of efficiency scores in the CNB that considered both accuracy and speed for all the cognitive domains, although being ecologically valid to reflect the real-life cognitive performance of the participants, could be confounded by a general lower processing speed. Future studies should examine how impaired processing speed may contribute to the executive dysfunction in ASD. Third, the PNC was not designed to study ASD and thus the clinical phenotyping methods were not specific to ASD, and the lack of severity measurement of the core features of ASD may account for the variability of comorbid psychopathology. Subdomains of EF such as inhibitory control and fluency were not considered in this study, which should be included in the future for more accurate representations of EF. The assessment of social cognition was limited to facial emotion recognition and differentiation, and did not capture higher-order ToM and mentalization processes. Fourth, our study did not include participants with intellectual disabilities, which has a prevalence of 30% within those with ASD (Lyall et al., 2017). The presence of intellectual disability is likely to have major implications on functioning and hence longitudinal outcomes of ASD (Anderson et al., 2014). The mean age of the study population was also relatively young (~12 years) and certain forms of psychopathology, such as depression and psychosis, tend to emerge at an older age. Future studies should examine the effects of intellectual disabilities and age.

## Conclusion

Impaired functioning and psychiatric comorbidities are common in ASD, where neurocognitive abilities are significant mediators. Our study suggests that in normal-IQ ASD, EF has a generalized effect, independent of other cognitive domains, across a wide spectrum of comorbid psychopathologies and functional levels in the disorder. EF may be a locus for intervention to improve clinical outcomes of this disabling disorder.

## Supplementary material

For supplementary material accompanying this paper visit https://doi.org/10.1017/S0033291722001787. click here to view supplementary material
