# Supplementary material for: Executive function as a generalized determinant of psychopathology and functional outcome in school-aged autism spectrum disorder: a case-control study
Source: Psychol Med. 2022 Aug 1;53(10):4788–98. doi: 10.1017/S0033291722001787 (PMC10388326; doi:10.1017/S0033291722001787)
Supplement: Supplementary file 1 [file S0033291722001787sup001.docx]

| **Table S1.** Descriptive statistics of the demographics and functional level of ASD and Non-ASD groups | | | | | | | | | |
| --- | --- | --- | --- | --- | --- | --- | --- | --- | --- |
|  |  | **ASD (N = 218)** | |  | **Non-ASD (N = 872)** | |  | **Statistical Comparison** | |
|  |  | **N / M** | **% / SD** |  | **N / M** | **% / SD** |  | **Statistics** | **p-value** |
| Age | | 12.267 | 3.024 |  | 12.334 | 3.302 |  | U = 94336 | 0.864 |
| Sex | |  |  |  |  |  |  | χ² = 0.000 | 1 |
|  | Female | 47 | 21.60% |  | 188 | 21.60% |  |  |  |
|  | Male | 171 | 78.40% |  | 684 | 78.40% |  |  |  |
| Race | |  |  |  |  |  |  | χ² = 0.163 | 0.922 |
|  | White | 167 | 76.60% |  | 675 | 77.40% |  |  |  |
|  | Black | 31 | 14.20% |  | 115 | 13.20% |  |  |  |
|  | Others | 20 | 9.20% |  | 82 | 9.40% |  |  |  |
| Education (in years) | |  |  |  |  |  |  |  |  |
|  | Subject | 5.436 | 2.913 |  | 5.607 | 3.275 |  | U = 94455 | 0.886 |
|  | Father | 15.068 | 2.706 |  | 14.655 | 2.68 |  | U = 79297 | 0.056 |
|  | Mother | 15.21 | 2.447 |  | 14.979 | 2.436 |  | U = 87749 | 0.228 |
| SES | | 0.370 | 0.820 |  | 0.379 | 0.774 |  | U = 92614.5 | 0.558 |
| Estimated IQ | | 103.404 | 16.149 |  | 104.820 | 16.003 |  | U = 90321.5 | 0.255 |
| C-GAS | | 66.335 | 13.634 |  | 81.253 | 10.921 |  | U = 36948.5 | < .001 |
| Note*.* ASD: Autism Spectrum Disorder; SES: Socioeconomic status; C-GAS: Children's Global Assessment Scale. IQ was estimated by the Wide Range Achievement Test 4 Reading Subscale | | | | | | | | | |

| **Table S2.** Pearson correlation coefficients between all variables | | | | | | | | | | | | | | | | | | | | |
| --- | --- | --- | --- | --- | --- | --- | --- | --- | --- | --- | --- | --- | --- | --- | --- | --- | --- | --- | --- | --- |
|  |  | **1** | **2** | **3** | **4** | **5** | **6** | **7** | **8** | **9** | **10** | **11** | **12** | **13** | **14** | **15** | **16** | **17** | **18** | **19** |
| 1 | ASD | — |  |  |  |  |  |  |  |  |  |  |  |  |  |  |  |  |  |  |
| 2 | Sex | 0 | — |  |  |  |  |  |  |  |  |  |  |  |  |  |  |  |  |  |
| 3 | Race - White | -0.008 | 0.04 | — |  |  |  |  |  |  |  |  |  |  |  |  |  |  |  |  |
| 4 | Race - Black | 0.012 | -0.075^*^ | -0.725^***^ | — |  |  |  |  |  |  |  |  |  |  |  |  |  |  |  |
| 5 | Race - Other | -0.003 | 0.031 | -0.592^***^ | -0.126^***^ | — |  |  |  |  |  |  |  |  |  |  |  |  |  |  |
| 6 | Age | -0.008 | -0.048 | 0.039 | -0.047 | -0.001 | — |  |  |  |  |  |  |  |  |  |  |  |  |  |
| 7 | Education | -0.021 | -0.044 | 0.052 | -0.058 | -0.006 | 0.983^***^ | — |  |  |  |  |  |  |  |  |  |  |  |  |
| 8 | SES | -0.005 | 0.076^*^ | 0.601^***^ | -0.64^***^ | -0.116^***^ | 0.05 | 0.068^*^ | — |  |  |  |  |  |  |  |  |  |  |  |
| 9 | Estimated IQ | -0.035 | 0.015 | 0.003 | 0.015 | -0.021 | 0.043 | 0.034 | -0.038 | — |  |  |  |  |  |  |  |  |  |  |
| 10 | Social Cognition Efficiency | -0.177^***^ | 0.126^***^ | 0.128^***^ | -0.164^***^ | 0.007 | 0.04 | 0.06^*^ | 0.191^***^ | -0.028 | — |  |  |  |  |  |  |  |  |  |
| 11 | Complex Reasoning Efficiency | -0.078^**^ | 0.026 | 0.236^***^ | -0.302^***^ | 0.012 | 0.028 | 0.053 | 0.287^***^ | -0.031 | 0.685^***^ | — |  |  |  |  |  |  |  |  |
| 12 | Memory Efficiency | -0.205^***^ | 0.044 | 0.026 | -0.06^*^ | 0.033 | 0.027 | 0.036 | 0.048 | -0.011 | 0.522^***^ | 0.466^***^ | — |  |  |  |  |  |  |  |
| 13 | Executive Efficiency | -0.109^***^ | 0.029 | 0.122^***^ | -0.16^***^ | 0.011 | -0.015 | 0.012 | 0.173^***^ | -0.019 | 0.519^***^ | 0.594^***^ | 0.41^***^ | — |  |  |  |  |  |  |
| 14 | Anxious-Misery | 0.289^***^ | -0.012 | -0.075^*^ | 0.036 | 0.065^*^ | -0.078^*^ | -0.077^*^ | -0.127^***^ | 0.018 | -0.097^**^ | -0.107^***^ | -0.048 | -0.078^*^ | — |  |  |  |  |  |
| 15 | Psychosis | 0.276^***^ | -0.046 | -0.129^***^ | 0.103^***^ | 0.066^*^ | -0.043 | -0.046 | -0.16^***^ | 0.025 | -0.158^***^ | -0.182^***^ | -0.088^**^ | -0.122^***^ | 0.831^***^ | — |  |  |  |  |
| 16 | Externalizing | 0.296^***^ | -0.098^**^ | -0.126^***^ | 0.107^***^ | 0.056 | -0.078^**^ | -0.078^**^ | -0.182^***^ | 0.028 | -0.131^***^ | -0.161^***^ | -0.08^**^ | -0.127^***^ | 0.684^***^ | 0.679^***^ | — |  |  |  |
| 17 | Fear | 0.247^***^ | 0.05 | -0.098^**^ | 0.07^*^ | 0.059 | -0.087^**^ | -0.087^**^ | -0.135^***^ | 0.006 | -0.137^***^ | -0.147^***^ | -0.095^**^ | -0.117^***^ | 0.741^***^ | 0.642^***^ | 0.518^***^ | — |  |  |
| 18 | P factor | 0.256^***^ | -0.048 | -0.127^***^ | 0.105^***^ | 0.061^*^ | -0.063^*^ | -0.065^*^ | -0.161^***^ | 0.03 | -0.129^***^ | -0.158^***^ | -0.054 | -0.101^***^ | 0.875^***^ | 0.96^***^ | 0.717^***^ | 0.692^***^ | — |  |
| 19 | C-GAS | -0.461^***^ | 0.019 | 0.05 | -0.064^*^ | 0.002 | -0.058 | -0.037 | 0.104^***^ | -0.009 | 0.214^***^ | 0.206^***^ | 0.172^***^ | 0.217^***^ | -0.482^***^ | -0.457^***^ | -0.509^***^ | -0.385^***^ | -0.466^***^ | — |
| Note. ASD: Autism Spectrum Disorder; SES: Socioeconomic status; WRAT: Wide Range Achievement Test-4; C-GAS: Children's Global Assessment Scale. For the ASD variable, “0” denotes non-ASD and “1” denotes ASD. IQ was estimated by the Wide Range Achievement Test 4 Reading Subscale. P-values were Bonferroni corrected for multiple comparison; * p < .05, ** p < .01, *** p < .001 | | | | | | | | | | | | | | | | | | | | |

| **Table S3.** Summary and comparison of goodness of fit statistics for the 3 structural equation models with and without estimated IQ as a covariate | | | | | | | | | | |
| --- | --- | --- | --- | --- | --- | --- | --- | --- | --- | --- |
|  |  | **df** | **χ²** | **p** | **CFI** | **RMSEA**  **[90% CI]** | **SRMR** | **R²** | **ΔR²** | **p** |
| Model 1 | | 5 | 28.525 | < .001 | 0.987 | 0.066  [0.044, 0.090] | 0.015 | 0.111 | - | - |
| Model 1a (controlled for estimated IQ) | | 5 | 23.361 | < .001 | 0.986 | 0.079  [0.051, 0.110] | 0.023 | 0.113 | 0.002 | 0.582 |
|  |  |  |  |  |  |  |  |  |  |  |
| Model 2 | | 16 | 64.925 | < .001 | 0.99 | 0.053  [0.040, 0.067] | 0.018 | - | - | - |
|  | Anxious - misery |  |  |  |  |  |  | 0.118 |  |  |
|  | Psychosis |  |  |  |  |  |  | 0.127 |  |  |
|  | Externalizing |  |  |  |  |  |  | 0.147 |  |  |
|  | Fear |  |  |  |  |  |  | 0.103 |  |  |
| Model 2a (controlled for estimated IQ) | | 18 | 73.568 | < .001 | 0.973 | 0.047  [0.031, 0.063] | 0.034 |  |  |  |
|  | Anxious - misery | - | - | - | - | - | - | 0.122 | 0.004 | 0.350 |
|  | Psychosis |  |  |  |  |  |  | 0.130 | 0.004 | 0.356 |
|  | Externalizing |  |  |  |  |  |  | 0.148 | 0.001 | 0.914 |
|  | Fear |  |  |  |  |  |  | 0.106 | 0.003 | 0.479 |
|  |  |  |  |  |  |  |  |  |  |  |
| Model 3 | | 4 | 10.974 | 0.027 | 0.996 | 0.040  [0.012, 0.069] | 0.011 | 0.264 | - | - |
| Model 3a (controlled for estimated IQ) | | 4 | 11.116 | 0.011 | 0.991 | 0.050  [0.021, 0.083] | 0.016 | 0.266 | 0.002 | 0.582 |
| Note. ASD: Autism Spectrum Disorder; C-GAS: Children’s Global Assessment Scale; CFI: Comparative Fit Index; RMSEA: Root Mean Square Error of Approximation; 90% CI: 90% Confidence Interval; SRMR: Standardized Root Mean Square Residual; controlled for sex, age, race, SES +/- IQ estimated by Wide Range Achievement Test-4 Reading Subscale. | | | | | | | | | | |

| **Table S4.** Summary of MANCOVA on neurocognitive efficiencies and psychopathology factors between ASD and Non-ASD groups with estimated IQ as covariate | | | | | | | | |
| --- | --- | --- | --- | --- | --- | --- | --- | --- |
| Neurocognitive efficiencies | **Multivariate Tests** | | | | | | | |
|  |  |  | **value** | **F** | **df1** | **df2** | **p** | **Partial η²** |
|  | ASD | Pillai's Trace | 0.057 | 16.383 | 4 | 1080 | < .001 | 0.031 |
|  | **Univariate Tests** | | | | | | |  |
|  |  | **Dependent Variable** | **SS** | **df** | **MS** | **F** | **p** | **Partial η²** |
|  | ASD | Social Cognition | 38.840 | 1 | 38.840 | 36.837 | < .001 | 0.031 |
|  |  | Complex Reasoning | 6.488 | 1 | 6.488 | 7.256 | 0.007 | 0.006 |
|  |  | Memory | 48.016 | 1 | 48.016 | 47.910 | < .001 | 0.042 |
|  |  | Executive Function | 16.895 | 1 | 16.895 | 13.332 | < .001 | 0.012 |
|  | Residuals | Social Cognition | 1141.914 | 1083 | 1.054 |  |  |  |
|  |  | Complex Reasoning | 968.313 | 1083 | 0.894 |  |  |  |
|  |  | Memory | 1085.401 | 1083 | 1.002 |  |  |  |
|  |  | Executive Function | 1372.427 | 1083 | 1.267 |  |  |  |
|  |  |  |  |  |  |  |  |  |
| Psychopathology factors | **Multivariate Tests** | | | | | | |  |
|  |  |  | **value** | **F** | **df1** | **df2** | **p** | **Partial η²** |
|  | ASD | Pillai's Trace | 0.127 | 31.471 | 5 | 1079 | < .001 | 0.066 |
|  | **Univariate Tests** | | | | | | |  |
|  |  | **Dependent Variable** | **SS** | **df** | **MS** | **F** | **p** | **Partial η²** |
|  | ASD | Anxious - Misery | 93.623 | 1 | 93.623 | 101.358 | < .001 | 0.085 |
|  |  | Psychosis | 80.874 | 1 | 80.874 | 92.539 | < .001 | 0.079 |
|  |  | Externalizing | 102.428 | 1 | 102.428 | 109.964 | < .001 | 0.092 |
|  |  | Fear | 64.359 | 1 | 64.359 | 72.414 | < .001 | 0.062 |
|  |  | P factor | 69.834 | 1 | 69.834 | 78.678 | < .001 | 0.068 |
|  | Residuals | Anxious - Misery | 1000.356 | 1083 | 0.924 |  |  |  |
|  |  | Psychosis | 946.488 | 1083 | 0.874 |  |  |  |
|  |  | Externalizing | 1008.783 | 1083 | 0.931 |  |  |  |
|  |  | Fear | 962.520 | 1083 | 0.889 |  |  |  |
|  |  | P factor | 961.264 | 1083 | 0.888 |  |  |  |
| Note. Results were controlled for sex, age, race, SES and IQ estimated by Wide Range Achievement Test-4 Reading Subscale. | | | | | | | | |

| **Table S5.** Mediation path estimates of the 3 structural equation models including IQ estimated by Wide Range Achievement Test-4 Reading Subscale as covariate as sensitivity analysis | | | | | | | | | | | | | |
| --- | --- | --- | --- | --- | --- | --- | --- | --- | --- | --- | --- | --- | --- |
| **Model 1a** | | **P factor** | | | | | | | | | | | |
|  |  | **Estimate [95% CI]** | | | | |  | **p** | | | | | |
| Direct effect | | | | | | | | | | | | | |
|  | ASD | 0.619 [0.475, 0.764] | | | | |  | < .001 | | | | | |
| Indirect effect | | | | | | | | | | | | | |
|  | ASD via Social Cognition Efficiency | 0.038 [0.010, 0.067] | | | | |  | 0.011 | | | | | |
|  | ASD via Complex Reasoning Efficiency | 0.027 [0.007, 0.059] | | | | |  | 0.027 | | | | | |
|  | ASD via Memory Efficiency | -0.001 [-0.017, 0.010] | | | | |  | 0.849 | | | | | |
|  | ASD via Executive Function Efficiency | 0.020 [0.005, 0.047] | | | | |  | 0.043 | | | | | |
|  |  |  | | | | |  |  | | | | | |
| **Model 2a** | | **Anxious-misery** | |  | **Psychosis** | |  | **Externalizing** | |  | **Fear** | |  |
|  |  | **Estimate [95% CI]** | **p** |  | **Estimate [95% CI]** | **p** |  | **Estimate [95% CI]** | **p** |  | **Estimate [95% CI]** | **p** |  |
| Direct effect | | | | | | | | | | | | | |
|  | ASD | 0.734 [0.588, 0.879] | < .001 |  | 0.677 [0.532, 0.821] | < .001 |  | 0.738 [0.882, 0.882] | < .001 |  | 0.593 [0.447, 0.740] | < .001 |  |
|  |  |  |  |  |  |  |  |  |  |  |  |  |  |
| Indirect effect | | | | | | | | | | | | | |
|  | ASD via Social Cognition Efficiency | 0.037 [0.009, 0.070] | 0.020 |  | 0.049 [0.020, 0.085] | 0.004 |  | 0.021 [0.051, 0.021] | 0.202 |  | 0.042 [0.013, 0.074] | 0.011 |  |
|  |  |  |  |  |  |  |  |  |  |  |  |  |  |
|  | ASD via Complex Reasoning Efficiency | 0.019 [0.003, 0.041] | 0.072 |  | 0.032 [0.005, 0.060] | 0.037 |  | 0.028 [0.005, 0.057] | 0.053 |  | 0.027 [0.004, 0.054] | 0.056 |  |
|  |  |  |  |  |  |  |  |  |  |  |  |  |  |
|  | ASD via Memory Efficiency | 0.008 [-0.024, 0.040] | 0.761 |  | 0.028 [-0.002, 0.059] | 0.089 |  | 0.025 [-0.008, 0.058] | 0.189 |  | 0.038 [0.006, 0.071] | 0.042 |  |
|  |  |  |  |  |  |  |  |  |  |  |  |  |  |
|  | ASD via Executive Function Efficiency | 0.026 [0.003, 0.049] | 0.032 |  | 0.035 [0.005, 0.064] | 0.026 |  | 0.039 [0.007, 0.071] | 0.028 |  | 0.037 [0.005, 0.069] | 0.047 |  |
|  |  |  |  |  |  |  |  |  |  |  |  |  |  |
|  |  |  |  |  |  |  |  |  |  |  |  |  |  |
| **Model 3a** | | **C-GAS** | | | | | | | | | | | |
|  |  | **Estimate [95% CI]** | | | | |  | **p** | | | | | |
| Direct effect | | | | | | | | | | | | | |
|  | ASD | -1.104 [-1.259, -0.947] | | | | |  | < .001 | | | | | |
| Indirect effect | | | | | | | | | | | | | |
|  | ASD via Social Cognition Efficiency | -0.010 [-0.045, 0.028] | | | | |  | 0.624 | | | | | |
|  | ASD via Complex Reasoning Efficiency | -0.020 [-0.056, 0.001] | | | | |  | 0.070 | | | | | |
|  | ASD via Memory Efficiency | 0.011 [-0.022, 0.048] | | | | |  | 0.537 | | | | | |
|  | ASD via Executive Function Efficiency | -0.028 [-0.064, -0.010] | | | | |  | 0.021 | | | | | |
| Note. ASD: Autism Spectrum Disorder; C-GAS: Children’s Global Assessment Scale; Results were controlled for sex, age, race and socioeconomic status, and IQ estimated by Wide Range Achievement Test-4 Reading Subscale, 95% bootstrap Confidence Interval, with Benjamini–Hochberg’s FDR method corrected for multiplicity.. | | | | | | | | | | | | | |


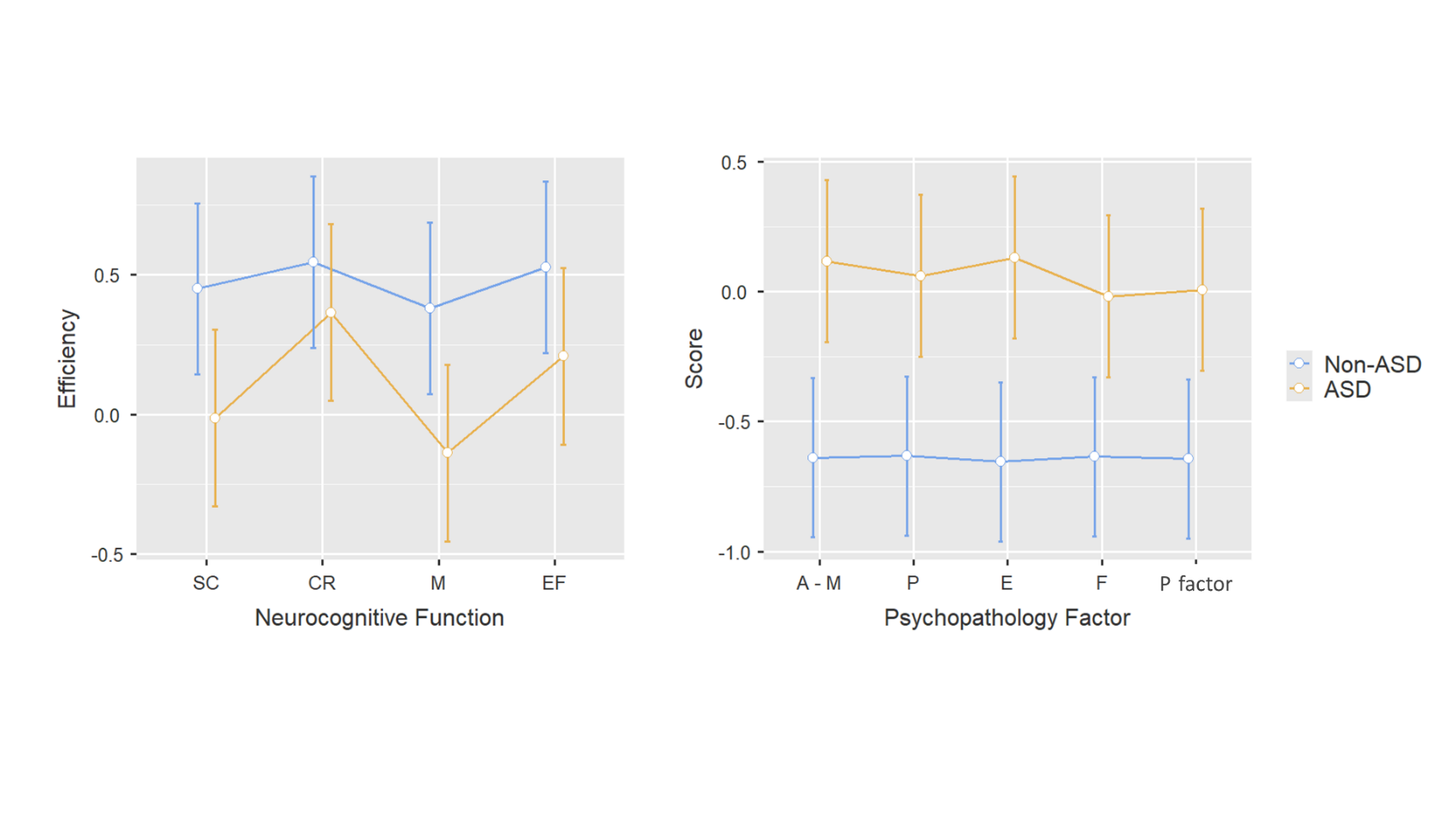
 Figure S1. Profiles of neurocognitive function efficiencies and psychopathology factors of participants with and without (non-) ASD, with error bars representing the 95% Confidence Interval. SC: Social Cognition Efficiency; CR: Complex Reasoning Efficiency; M: Memory Efficiency; EF: Executive Function Efficiency. A – M: Anxious – Misery; P: Psychosis; E: Externalizing; F: Fear.


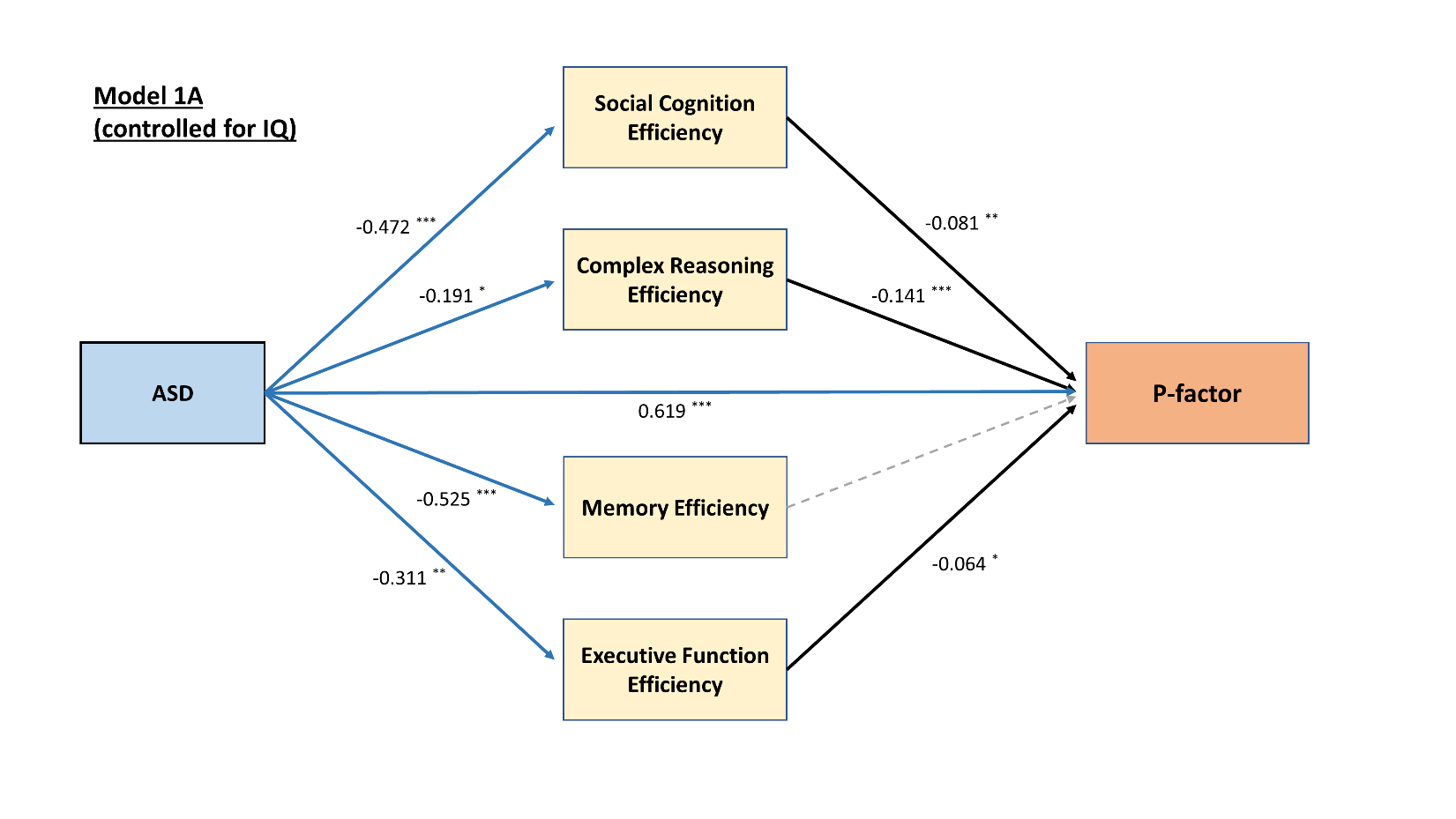
Figure S2. Structural regression model for the effect of ASD on the P factor, mediated by neurocognitive function efficiencies with additional control for IQ as covariate. Presented estimates are beta coefficients, with statistically significant paths shown in solid lines. *p < .05, **p < .01, ***p < .001.


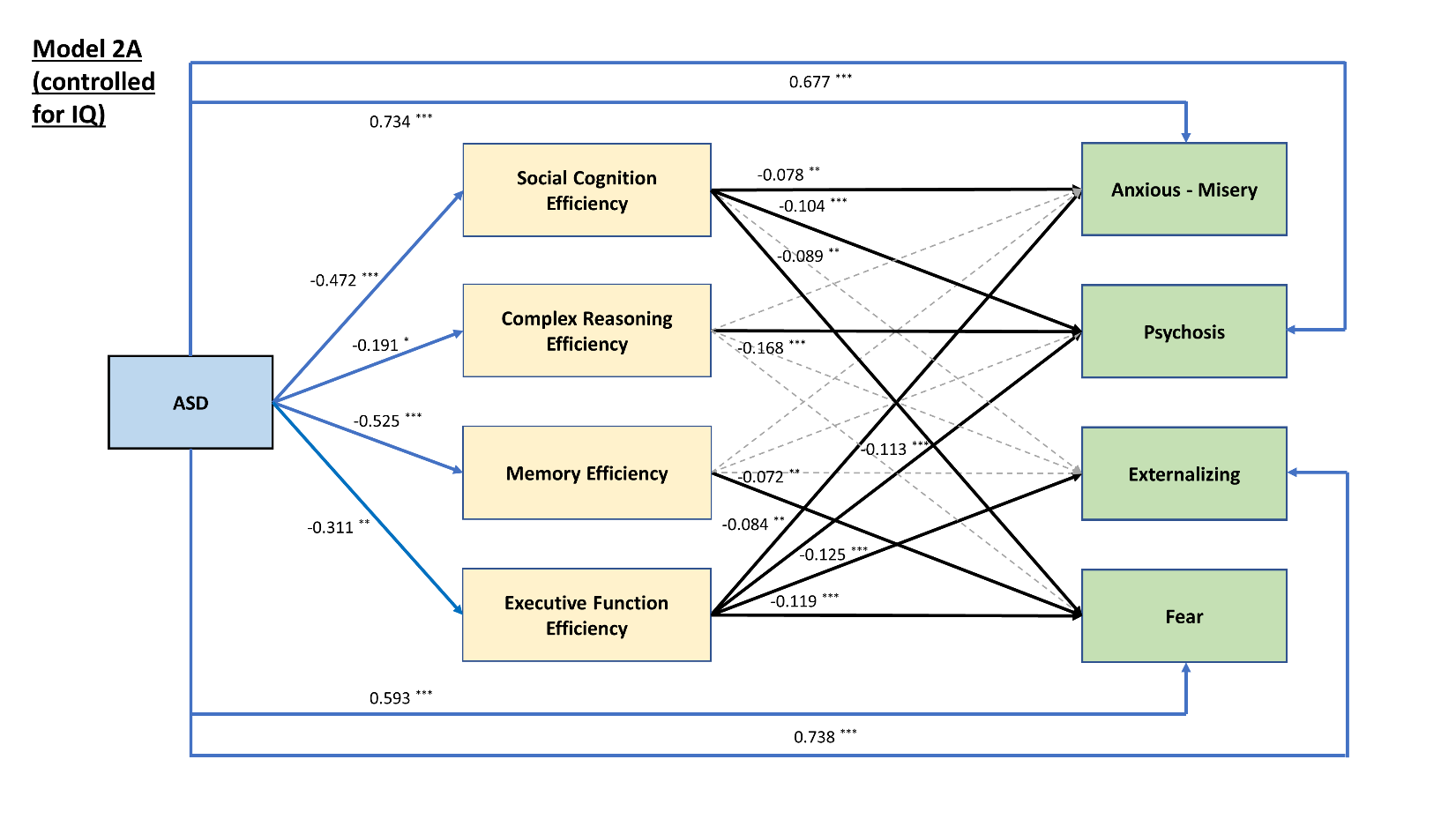
Figure S3. Structural regression model for the effect of ASD on the four psychopathology factors, mediated by neurocognitive function efficiencies with additional control for IQ as covariate. Presented estimates are beta coefficients, with statistically significant paths shown in solid lines. *p < .05, **p < .01, ***p < .001.


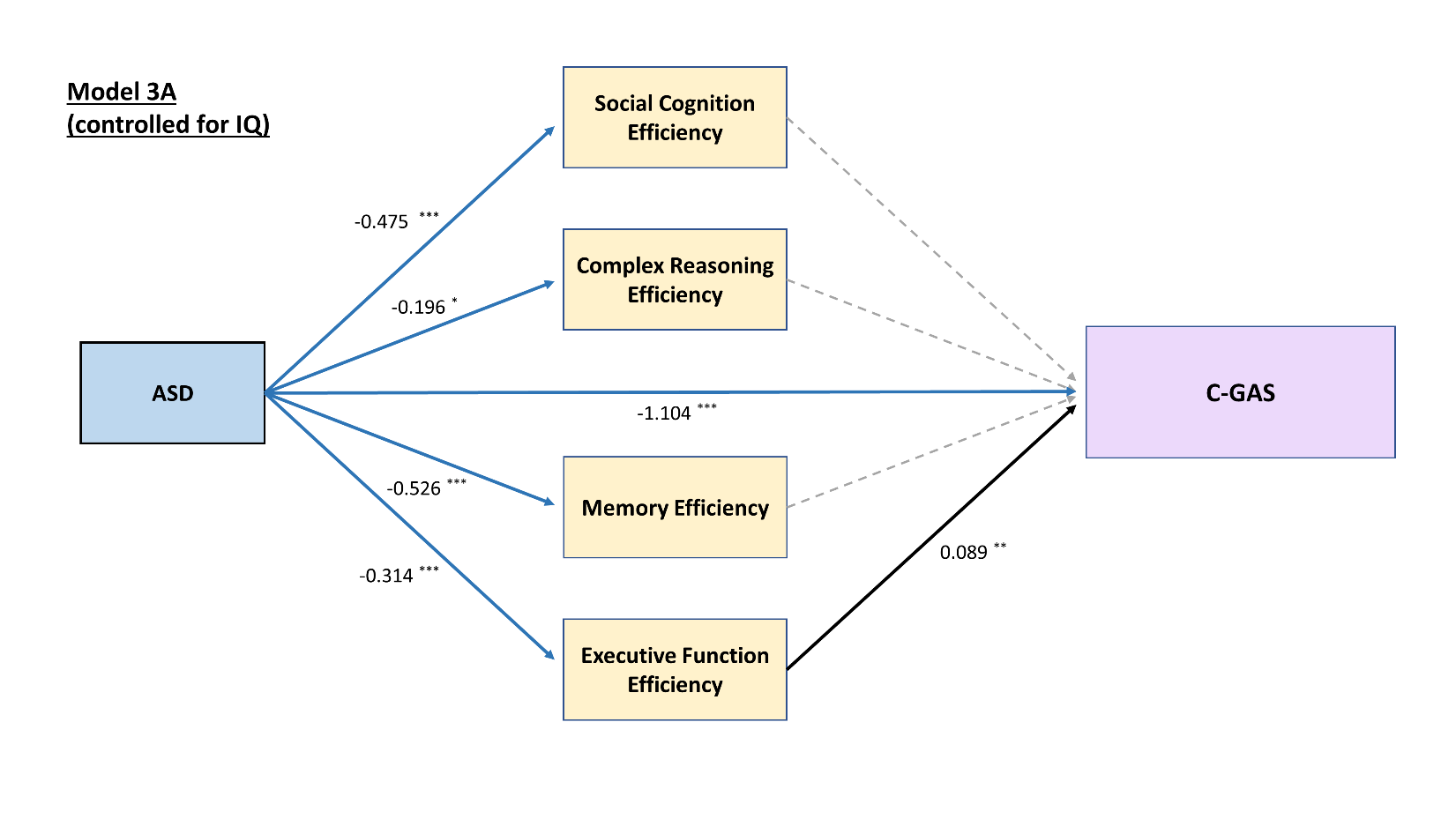
Figure S4. Structural regression model for the effect of ASD on functional level, as measured by the Children’s Global Assessment Scale (C-GAS) mediated by neurocognitive function efficiencies with additional control for IQ as covariate. Presented estimates are beta coefficients, with statistically significant paths shown in solid lines. *p < .05, **p < .01, ***p < .001.
